# Nutritional Supplementation to Increase Influenza Vaccine Response in Children Living With HIV: A Pilot Clinical Trial

**DOI:** 10.3389/fped.2022.919753

**Published:** 2022-07-19

**Authors:** Talía Sainz, Inmaculada Casas, Mónica González-Esguevillas, Luis Escosa-Garcia, María Ángeles Muñoz-Fernández, Luis Prieto, María José Gosalbes, Nuria Jiménez-Hernández, José Tomas Ramos, María Luisa Navarro, María José Mellado, Sergio Serrano-Villar, Cristina Calvo

**Affiliations:** ^1^Servicio de Pediatría, Hospital Universitario La Paz and IdiPAZ, Madrid, Spain; ^2^Red de Investigación Traslacional en Infectología Pediátrica (RITIP), Madrid, Spain; ^3^Centro de Investigación Biomédica en Red en Enfermedades Infecciosas (CIBERINFEC), Instituto de Salud Carlos III, Madrid, Spain; ^4^Respiratory Virus and Influenza Unit, Instituto de Salud Carlos III, Madrid, Spain; ^5^Centro de Investigación Biomédica en Red en Epidemiología y Salud Pública (CIBERESP), Madrid, Spain; ^6^Laboratorio de InmunoBiología Molecular Hospital General Universitario Gregorio Marañón e IISHGM, Madrid, Spain; ^7^Hospital 12 de Octubre, Madrid, Spain; ^8^Universidad Complutense de Madrid (UCM), Madrid, Spain; ^9^Área Genómica y Salud, Fundación Para el Fomento de la Investigación Sanitaria y Biomédica (FISABIO), Valencia, Spain; ^10^Servicio de Pediatría, Hospital Clinico San Carlos and IdISSC, Madrid, Spain; ^11^Unidad de Investigación Materno-Infantil Familia Alonso (UDIMIFFA), IISGM, Servicio de Pediatría, Hospital General Universitario Gregorio Marañón e IISHGM, Madrid, Spain; ^12^Servicio de Enfermedades Infecciosas, Hospital Universitario Ramón y Cajal, and IRYCIS, Madrid, Spain

**Keywords:** HIV, influenza vaccine response, children, microbiota, immunoactivation

## Abstract

**Aims:**

Vaccine response is poor among children living with HIV. The gut microbiota has been identified as a potential target to improve vaccine immunogenicity, but data are scarce in the context of HIV infection.

**Methods:**

Pilot, double-blind, randomized placebo-controlled trial in which 24 HIV-infected children were randomized to receive a mixture of symbiotics, omega-3/6 fatty acids, and amino acids or placebo for 4 weeks, each in combination with ART, and were then immunized against influenza. Vaccine response and safety of the nutritional supplementation were the primary outcomes.

**Results:**

Eighteen HIV-infected children completed the follow-up period (mean age 11.5 ± 4.14 years, 61% female). The nutritional supplement was safe but did not enhance the response to the influenza vaccine. A 4-fold rise in antibody titers was obtained in only 37.5% of participants in the intervention arm vs. 40% in the placebo. No immunological or inflammatory predictors of vaccine response were identified.

**Conclusions:**

In this exploratory study, a 4-week course of symbiotics did not increase influenza vaccine immunogenicity in HIV-infected children. Larger studies are warranted to address the potential of modulating the microbiome in children living with HIV.

## Summary

Pilot randomized clinical trial addressing the impact of a 4 weeks nutritional intervention targeting gut dysbiosis on the response to influenza immunization among HIV-infected children on ART. Despite mild changes on microbiota composition, the intervention did not affect influenza vaccine response.

## Introduction

Despite achieving and maintaining viral suppression under antiretroviral treatment (ART), immunological restoration is not complete during HIV infection (1). Persistent inflammation and immunoactivation have been described both among adults and children and are a matter of concern (1–3). In HIV-infected children, innate and adaptive abnormalities (2) probably contribute to the reduction in the magnitude and durability of vaccine response (4–7). This impaired immunity leads to an increased rate of vaccine-preventable diseases in this population, greater severity and high progression-rate of vaccine-preventable infections, from measles to pneumococcal disease (8–11). Among other immunizations, influenza is amongst the vaccines with lower immunogenicity in people living with HIV (PLWHIV) (5, 12–15).

Based on the interactions that occur among bacteria and the immune system at the intestinal mucosa, the gut microbiome has been identified as a potential target for interventions aimed at increasing vaccine immunogenicity (16). Changes in the composition and function of the microbiome in PLWHIV are thought to be explained by the depletion of lymphocyte populations during acute infection at the gut mucosa, only partially restored by ART (17, 18). These abnormalities, together with microbial translocation and viral persistence, may contribute to the persistent immune dysfunction associated to chronic HIV infection (17, 19). The potential of targeting the gut microbiome in this population is therefore of interest. Studies describing the extent of gut dysbiosis among HIV-infected children have arisen controversial results (20–22). Clinical trials aiming at modulating the gut microbiome via nutritional supplementation, mostly in adults, have achieved only mild changes in the ecosystem and partial immunological effects (22–26). Because the assembling and establishment of human microbiome occurs during the first years of life (27), the potential to modulate and consequently impact the bacteria-immune system interplay might be therefore higher during childhood. However, attempts to target the microbiome in the unique population of perinatally HIV-infected children are scarce.

On the basis that the gut microbiota-immune system interactions likely influence vaccine response, we aimed to assess the potential impact of a nutritional supplementation aimed at modulating the dysbiosis of perinatally HIV-infected children on the response to influenza vaccine.

## Methods

### Study Design and Participants

Pilot, double blind, randomized, placebo-controlled study. Vertically HIV-infected children and adolescents aged 6–18 years, on stable ART for at least 6 months and with CD4+ T-cell counts ≥350 cells/μL, were enrolled at the HIV clinics of four Hospitals in Madrid, Spain, October 2013 to November 2014. Exclusion criteria included acute or chronic infections other that HIV, antibiotic treatment in the previous 3 months, as well as presence of other chronic conditions requiring medication. Participants were randomly assigned to receive a nutritional supplementation or placebo by a computer-generated randomized number system in blocks, daily for 4 weeks, and were then immunized against influenza infection. Peripheral blood and fecal samples were collected at baseline and after the 4-weeks intervention (±7 days). Serum samples for measurement of antibody titers against influenza were obtained immediately before and 3 months after immunization. Participants received the intramuscular quadrivalent influenza vaccine, according to recommendations by the Spanish Ministry of Health, for active immunization for the prevention of influenza A and B virus (Fluarix®, Vaxigrip tetra®). The participating clinicians, the laboratory personnel, and the study participants and family were blind to the assigned patient group.

The study protocol was approved by the Independent Ethics Committee at all participating Institutions (approval number 173/13) and all parents and participants above 12 years of age provided written informed consent/assent. Full details on the study protocol have been previously published (22).

### Nutritional Intervention

A 20 mg powder combination of a specifically design nutritional supplementation containing pre/probiotics, oligosaccharides, glutamine, AM3, and vitamin D (PMT25341) (23) or placebo (skimmed milk powder) was administered daily. Both products were prepared by Nutricion Médica, S.L. in identically appearing sealed envelopes.

### Laboratory Assessments

Fasting blood samples were drawn at baseline and at post-intervention for real-time measurements of plasma HIV-1 viral load and immunological studies, by standard flow cytometric methods, and included T-cell activation (HLA-DR^+^/CD38^+^), senescence (CD28^−^CD57^+^) and exhaustion (PD-1) markers. Stained cells were run on a Gallios flow cytometer (Beckman Coulter, Inc., Münster, Germany), and data analyzed using Kaluza software (Beckman Coulter, Inc, Münster, Germany). A panel of inflammatory biomarkers and cytokines including IL-17A, IL-10, IL-6, and IP-10 was determined using a multiplex immunoassays by Invitrogen™ ProcartaPlex™ (ThermoFisher) and ELISAs for IL-7 (R&D IL-7 Quantikine HS ELISA Kit), sCD14 (Quantikine ELISA Kit DC140, RD), and zonuline (Abyntek Biopharma S.L. ELISA Kit). The plasma kynurenine to tryptophan ratio (KT ratio) was determined by mass spectrometry, using a liquid chromatography system consisting of a degasser, binary pump and autosampler (1290 Infinity, Agilent Technologies, Santa Clara, CA, USA) coupled to a triple quadrupole mass spectrometer (6460, AgilentTechnologies).

A serum sample of every participant was sent to the Pediatric HIV BioBank-HGUGM processed and stored at −80° using standard procedures for subsequent determinations. Antibody titers against the three viral haemagglutinin (HA) components of influenza vaccine used in seasons 2013–2014 and 2014–2015, (A/H1N1, A/California/07/09, A/H3N2, A/Victoria/361/2011 and A/Texas/50/2012, B/Massachusetts/2/12), and were determined by Haemagglutination Inhibition Assay, using both guinea-pig and turkey erythrocytes. All samples were studied simultaneously in order to prevent titer differences between assays. A 4-fold rise in titer was interpreted as significant.

Fecal samples were collected in sterile tubes with RNAlater (Life Technologies) and stored at −80°C until use. Total DNA was extracted and the V3-V4 region of the 16S rRNA gene were amplified from total DNA. The amplicon libraries were constructed following Illumina instructions, quantified with Qubit Fluorometer (ThermoFisher, Waltham, MA, USA) and sequenced using the Kit v3 (2 × 230 cycles) in a MiSeq platform (Illumina) as previously published (22).

### Statistical Analysis

We used the Mann-Whitney *U* test for the between-group comparisons of continuous variables and Wilcoxon signed-rank matched-pairs test to evaluate differences in numerical outcomes between time-points. Fold changes between baseline and week four measurements were calculated to analyze correlations between inflammatory and T-cell biomarkers and vaccine response. Statistical analysis was performed using Stata v17.0 (StataCorp LP, College Station, TX) and Prism v.7.0, GraphPad, Inc., La Jolla, CA).

## Results

Twenty-four vertically HIV-infected children and adolescents on ART and virologically suppressed were recruited and randomized, but only 18 completed the follow-up period. Two patients withdrew during follow-up period and four were not immunized within the per protocol ±7 days period after supplementation. Eight patients in the intervention arm and 10 in the placebo group were immunized against influenza and had available serum samples. Median age was 11.5 ± 4.14 years, 61% were female. Patients on the intervention group were younger and had a higher CD4 nadir, but no other significant differences at baseline in terms of immunological or inflammatory biomarkers, including CD4 counts (Table 1).

**Table 1:** Clinical characteristics and laboratory parameters of study participants.

	**Placebo *N* = 10**	**Nutritional intervention *N* = 8**	***P***
Female (*N*, %)	6 (60)	5 (62.5)	1.000
Age (years), mean (SD)	13.6 (3.5)	9 (3.5)	**0.033**
Black (*N*, %)	4 (40)	4 (50)	0.772
CD4 count (cells/mm^3^)	628 [486–736]	692 [517–861]	0.447
CD4/CD8 ratio	1.45 [0.75–1.93]	1.81 [1.36–1.90]	0.447
CD4 Nadir (cells/mm^3^)	333 [206–376]	519 [410–1,086]	**0.007**
PI based ART (*N*, %)	7 (70)	7 (87.5)	0.588
Lopinavir/ritonavir	2 (20)	6 (75)	
Atazanavir/ritonavir	2 (20)	0 (0)	
Darunavir/ritonavir	1 (10)	1 (12.5)	
NNRTI based ART (*N*, %)	3 (30)	1 (12.5)	0.543
Efavirenz	3 (30)	1 (12.5)	
INI based ART (*N*, %)	2 (20)	0 (0)	0.892
Raltegravir	2 (20)	0 (0)	
Time on ART (years)	12.8 (8.6–15.5)	8.6 (8.2–13.4)	0.067
HLADR+CD38+CD8 T cells	1.9 [0.96–3.77]	3.28 [1.66–9.17]	0.248
CD57+CD28- CD8 T cells	17 [11–25]	28 [22–32]	0.248
Interleukin 6	0.6 [0.29–1.23]	0.33 [0.10–0.67]	0.306
Interleukin 7	114.6 [96–128]	84.9 [43.5–127.5]	0.204
IP-10	47.2 [31.7–67.9]	43.5 [36.9–62.4]	1.000
sCD14	2.13 [2.0–2.2]	2.33 [1.7–2.5]	0.328
zonulin	4.3 [2.7–5.2]	3.9 [1.4–8.8]	0.922
KT ratio	320 [263–423]	296 [253–395]	0.614
4-fold increase antibody titers against Influenza A H1N1, *N* (%)	4 (40)	3 (37.5)	1.000
4-fold increase antibody titers against Influenza A H3N2, *N* (%)	1 (10)	0 (0)	0.923
4-fold increase antibody titers against Influenza B, *N* (%)	2 (20)	1 (12.5)	0.547

*ART, antiretroviral treatment; IP-10, Interferon gamma-induced protein; NNRTI, non-nucleoside reverse transcriptase analogs; INI, integrase inhibitors; PI, protease inhibitors; sCD14, soluble CD14; KT ratio, kynurenine to tryptophan ratio*.

*Statistical test used: Mann-Whitney U/Wilcoxon signed rank test/Fisher's exact test*.

*Bold indicates statistically significant (p < 0.05)*.

Reported adherence was good over the study period, with no tolerance issues, and no adverse events were registered.

**Figure 1:**
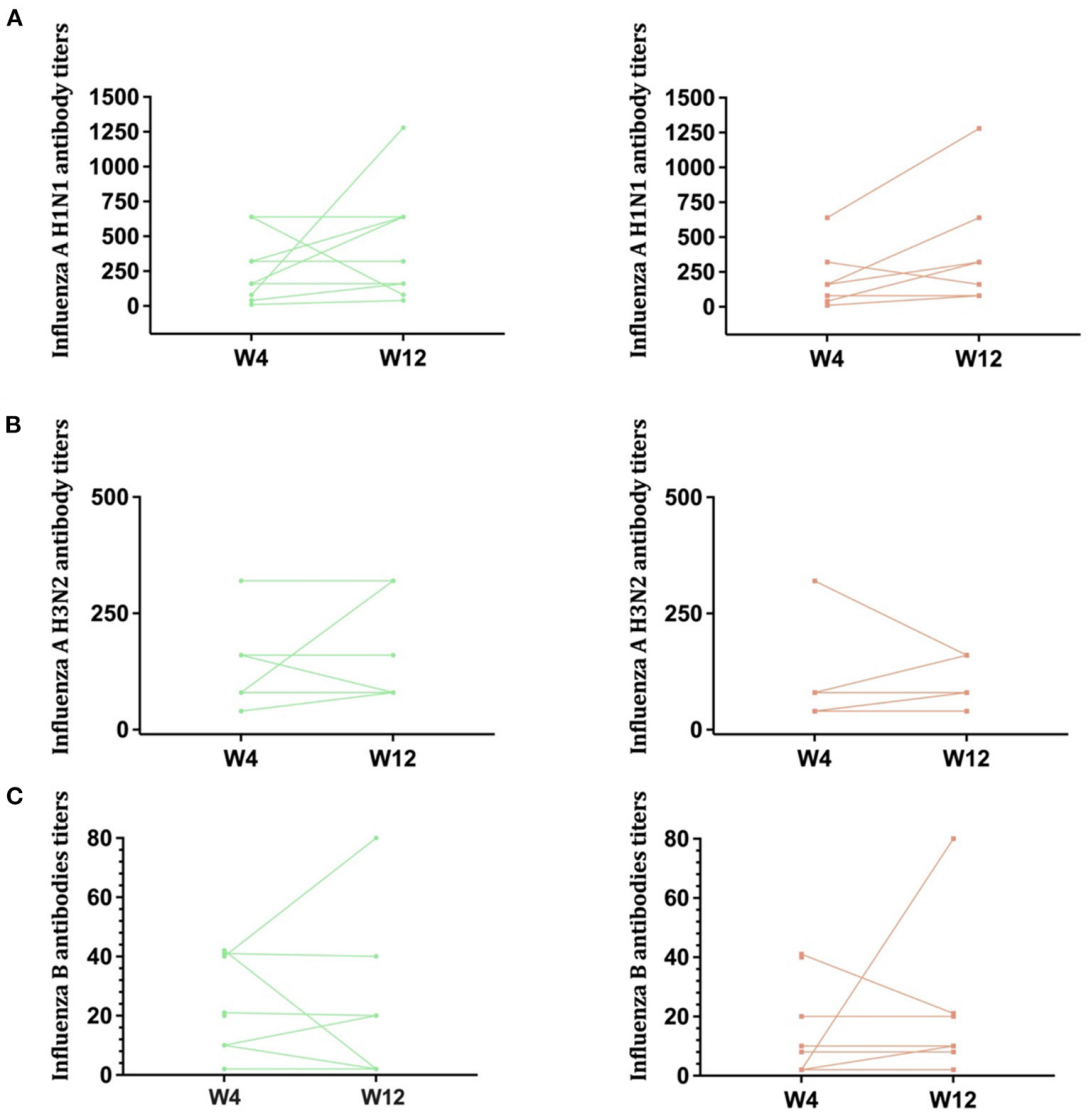
Antibody titers against influenza. Antibody titers against Influenza A H1N1 **(A)**, Influenza A H3N2 **(B)**, and Influenza B **(C)** are shown in this figure, according to randomization group (Left: placebo, Right: intervention arm). Lines represent individual trajectories.

Response to all components of the trivalent vaccine were poor. For the H1N1 component, a 4-fold rise in antibody titers after immunization was obtained in 3/8 (37.5%) of participants in the intervention arm vs. 4/10 (40%) in the placebo group. For the H3N2 component and for Influenza B responses were even lower (Table 1). Individual trajectories of participants serologies according to intervention arm are shown in Figure 1.

Results regarding fecal microbiota structure in comparison with uninfected controls have been previously published (22). In summary, the intervention did not lead to clear differences on alpha diversity. The differences in beta diversity present at baseline between groups disappeared after the intervention, suggesting that PMT25341 attenuated the compositional changes in the microbiota associated with HIV infection. However, changes over time within the groups were non-significant, and the nutritional supplementation did not achieve a significant increase in CD4 counts or any immunological marker. IL-6, IL-7, IL-10, Zonulin, Intestinal Fatty Acid Binding Protein, soluble CD14 (sCD14), or the KT ratio remain stable over time, with no differences between study arms (26).

No associations were found between the influenza antibody titers and the CD4/CD8 ratio, which has been suggested as a marker of immune reconstitution and vaccine response among people living with HIV (28, 29). None of the studied immune activation/senescence markers or the inflammatory and microbial translocation markers was identified as a predictor of vaccine response, including the KT ratio that has been shown to predict vaccine immunogenicity among adults living with HIV (29) (data not shown).

## Discussion

Results from this pilot study confirm that influenza vaccine response is extremely poor among HIV-infected children, with H1N1 being the component that elicits the most intense response. Despite being safe, a 4-week nutritional intervention failed to improve vaccine immunogenicity. These results suggest that targeting the gut microbiota might require more powerful or longer interventions, if aiming at achieving systemic effects.

HIV-infected children are at heightened risk for severe influenza illness (6, 7, 14), and immunization is recommended in most guidelines, even when immunogenicity seems particularly low in this population according to most series (5, 14). In the design of an exploratory trial, we understood this fact may have maximized our possibilities to achieve an effect, as the margin of improvement is greater for influenza vaccine compared to more immunogenic vaccines. However, the fact that no changes in CD4, CD8 or activation markers were observed (26) also supports the hypothesis of the shortness of the intervention, as T cell subpopulations typically evolve slowly. In the study by Cahn et al. that showed lower CD4 decline and a significant decrease of the immune activation markers in ART-naïve adults, probiotics were administered over a 52 weeks period (25). The fact that, for security reasons, a basal CD4 T cell count above 350 cell/mm was required for inclusion, may have excluded from participation those patients with potentially a greater margin of benefit. However, our previous experience using this nutritional supplement, designed to include components suggested to enhance gut epithelial barrier integrity, as well as stimulate immune recovery, precisely among ART naïve adults with CD4 < 350 counts/uL, and for 48 weeks, showed no clear immunological benefits in addition to ART (23). Modulating an established ecosystem might require additional interventions to the solely nutritional supplementation (30, 31), and the optimal supplements are still to be defined. Vaccine response was in fact extremely poor in our study compared to other series addressing also influenza immunization in children living with HIV (14, 15). This fact is even more shocking taking into account the good immunological status of the children included in this pilot trial, as previous studies have suggested that immunological status determines vaccine response (7, 32). The identification of predictors of vaccine response has been identified as an urgent need for this population that grows with the virus (32). Markers available to routine clinical practice would allow us to personalize vaccination schedules. In PLWHIV, both the CD4/CD8 ratio and the KT ratio, an indirect marker of the activity of the Indoleamine 2,3-dioxygenase-1 (IDO) that catabolizes tryptophan (T) to kynurenine (K), have been suggested to predict vaccine immunogenicity (29). None of these markers correlated here with vaccine response, and none improved due to the intervention, in line with the fact that vaccine response was not modified either.

Sample size is the main limitation of this exploratory trial, as the study might be underpowered to detect any differences. There were some unexpected differences between both groups, with younger patients with higher CD4 nadir in the intervention group. Despite the good immunological situation of all participants, the fact that IgG levels were unmeasured at baseline is also a limitation. The inclusion of patients with CD4 > 350 cell/mm impairs us to extrapolate results to immunological non-responders, which may be the population that hypothetically benefits most from an intervention addressing immune dysfunction. No adverse events occurred during the study period. Despite reported adherence was good, compliance with an additional treatment could have been suboptimal. Adherence issues are common among children and adolescents, and should be balanced when attempting to design strategies to improve quality of life in PLWHIV, especially during childhood and adolescence. Although adherence was reinforced during the trial, to what extent it may have affected the lack of differences found remains unknown.

To our knowledge, this exploratory trial is the first exploring the effect of a dietary intervention to improve vaccine response among vertically HIV-infected children. Response to influenza immunization, poor among participants, was not enhanced by means of pre/probiotic supplementation. Further studies and innovative approaches are required to address the potential transferability to clinical settings of microbiota modulation as a useful tool to improve the health of children living with HIV.

## Data Availability Statement

The datasets presented in this study can be found in online repositories. The names of the repository/repositories and accession number(s) can be found below: European Bioinformatics Institute (http://www.ebi.ac.uk/) database (accession number: PRJEB35283, secondary accession: ERP119574).

## Ethics Statement

The studies involving human participants were reviewed and approved by Hospital Gregorio Marañón Ethics Committee and all participating Hospitals, Madrid, Spain. Written informed consent to participate in this study was provided by the participants' legal guardian/next of kin.

## Author Contributions

TS: study conception and design, funding acquisition, recruitment, coordination, data analysis, and draft and final manuscript writing. IC: methodology, laboratory determinations, analysis and interpretation, funding acquisition, and writing. MG-E: laboratory determinations, analysis, and interpretation. LE-G and MM: recruitment and data collection at Hospital La Paz. MM-F: study design, coordination, funding acquisition, and writing. LP: recruitment and data collection at Hospital and H Getafe. MG: study design, laboratory analysis, statistical analysis and interpretation, and writing. NJ-H: sequencing and laboratory analysis. JR: recruitment and data collection at Hospital, and H. Clínico. MN: study coordination, recruitment and data collection at Hospital, and H Gregorio Marañón. SS-V: study conception and design, statistical analysis, interpretation, and writing. CC: conceptualization, study coordination, funding acquisition, and writing. All authors contributed to the article and approved the submitted version.

## Funding

This work was funded by the Instituto de Salud Carlos III-Fondos FEDER (grant number CB21/17/00025), Acción Estratégica en Salud (PI13/0422, PI17/01283, PI18/00154, and PI18CIII/00009). TS and SS-V have been funded by the Instituto de Salud Carlos III-Fondos FEDER (BA21/00022 and BA21/00017). The funding bodies did not have a role in the design or conduct of the study, the analysis and interpretation of the results, and the writing of the report or the decision to publish.

## Conflict of Interest

The authors declare that the research was conducted in the absence of any commercial or financial relationships that could be construed as a potential conflict of interest.

## Publisher's Note

All claims expressed in this article are solely those of the authors and do not necessarily represent those of their affiliated organizations, or those of the publisher, the editors and the reviewers. Any product that may be evaluated in this article, or claim that may be made by its manufacturer, is not guaranteed or endorsed by the publisher.
